# Problematic Analysis and Inadequate Toxicity Data in Phase III Apatinib Trial in Gastric Cancer

**DOI:** 10.1200/JCO.2016.67.3889

**Published:** 2016-08-15

**Authors:** Sheng Zhang

**Affiliations:** Sheng Zhang, Shanghai Cancer Center, Fudan University, Shanghai, People’s Republic of China

## To the Editor:

I have concerns about the reported results in the recent apatinib trial by Li et al^1^ in Chinese patients with gastric cancer.

In superiority trials, intention-to-treat (ITT) analyses that include all patients according to the allocated treatment are standard. This can lead to a conservative estimate of effect size and avoid bias associated with nonrandom loss of participants; however, excluding patients who did not start treatment or who stopped treatment during the trial can lead to biased estimates. This ITT analysis has been required by CONSORT statement, and the pitfalls of not performing ITT analysis have been documented previously.^2^ In the current study, five patients in the experimental arm and one patient in the placebo arm were excluded from the ITT analyses, which may exaggerate the modest survival benefit observed.^1^

Toxicity is one crucial aspect of clinical trial reporting. In a previous phase II trial that used apatinib for treatment of 25 patients with breast cancer, the dose of 750 mg once per day resulted in substantial toxicities: a dose delay of at least one cycle with dose reduction occurred in 84% of patients. Almost all patients experienced grade ≥ 3 toxicity, and treatment-related death occurred in two patients.^3^ Because of the heavy toxicity of apatinib, the dose was reduced to 500 mg once per day in further studies. In the current study, Li et al^1^ concluded that a dose of 850 mg once per day is tolerable and acceptable. Reasons for this discrepancy are not clear; however, even in the current trial, of the 40 patients that discontinued apatinib treatment, 22 patients (55%) stopped treatment as a result of toxicity, and dose reduction occurred in 21% patients who finished apatinib treatment.^1^ What is the total dose for patients who receive treatment with apatinib? What is the mean dose for these patients? Is there any treatment-related death? Because apatinib has been approved by the China Food and Drug Administration on the basis of the results of this trial and apatinib will now become commercially available in China, detailed clarification and explanation of the toxicity data remain essential for patient safety and evaluation of benefit versus risk, although the toxicity profile cannot be directly compared between different trials.

One good way to evaluate the efficacy and safety burden is quality-of-life data; however, the rate of compliance for responding to quality-of-life questionnaires in the apatinib group was surprising low (7.7%).^1^ This may also undermine the validity of the corresponding results. Therefore, I question the conclusion of the phase III study of apatinib in patients with gastric cancer by Li et al.^1^
